# Theoretical isotopic fractionation of magnesium between chlorophylls

**DOI:** 10.1038/s41598-017-07305-6

**Published:** 2017-08-01

**Authors:** Frédéric Moynier, Toshiyuki Fujii

**Affiliations:** 1 Institut de Physique du Globe de Paris, Sorbonne Paris Cité, Université Paris Diderot, CNRS, F-75005 Paris, France; 2 Institut Universitaire de France, Paris, France; 3 Division of Sustainable Energy and Environmental Engineering, Graduate School of Engineering, Osaka University, 2-1 Yamadaoka, Suita, Osaka 565-0871 Japan

## Abstract

Magnesium is the metal at the center of all types of chlorophyll and is thus crucial to photosynthesis. When an element is involved in a biosynthetic pathway its isotopes are fractionated based on the difference of vibrational frequency between the different molecules. With the technical advance of multi-collectors plasma-mass-spectrometry and improvement in analytical precision, it has recently been found that two types of chlorophylls (*a* and *b*) are isotopically distinct. These results have very significant implications with regards to the use of Mg isotopes to understand the biosynthesis of chlorophyll. Here we present theoretical constraints on the origin of these isotopic fractionations through *ab initio* calculations. We present the fractionation factor for chlorphyll a, b, d, and f. We show that the natural isotopic variations among chlorophyll *a* and *b* are well explained by isotopic fractionation under equilibrium, which implies exchanges of Mg during the chlorophyll cycle. We predict that chlorophyll *d* and *f* should be isotopically fractionated compared to chlorophyll *a* and that this could be used in the future to understand the biosynthesis of these molecules.

## Introduction

Magnesium is a ubiquitous element in nature and has three naturally occurring stable isotopes, ^24^Mg, ^25^Mg and ^26^Mg, with relative abundance of 78.99%, 10.00% and 11.01%, respectively. Magnesium also plays an essential role in a wide range of fundamental biological and non-biological reactions in the geological and biological system.

Chlorophylls are the green pigments, which allow phytoautotrophs to use light energy to produce organic matter by photosynthesis. Chlorophylls are formed of a Mg atom embedded in a porphyrin or chlorin ring^1, 2^. Chlorophylls can take several forms, with chlorophyll *a* the main pigment used by almost all oxygenic (oxygen-evolving) photosynthetic organisms. In addition to chlorophyll *a* (chl *a*), some organisms use accessory pigments: chlorophyll *b* (chl *b*) in green algae, and higher plant antennas, chlorophyll *c* (chl *c*) in certain algae, chlorophyll *d* (chl *d*) in some cyanobacteria and the recently discovered chlorophyll *f* (chl *f*) in some cyanobacteria^3^. In addition, there are a number of bacteriochlorophylls (BChl *a* through *g*) found in anoxygenic (non-oxygen evolving) photosynthetic bacteria^2, 4^.

Biological processes are widely recognized to have the ability to fractionate isotopes and create isotopic fractionation wherein the isotopic ratio of the product is different to that of the starting material (e.g. Zn, Fe, and Cu isotopic fractionation between plants and soils refs 5–10). Two studies found that Mg in cyanobacterial chl *a* is isotopically fractionated from the culture medium in which the cyanobacteria were grown and chl *a* and chl *b* have different isotopic compositions^11, 12^. They found that the ^26^Mg/^24^Mg of chl *a* was 0.43 ± 0.15‰ heavier than of chl *b*. These results have very important geochemical and biological implications: 1) Incorporating magnesium into chl *a* fractionates Mg isotopes and therefore the distribution of Mg isotopes in fossils may be used to search for the presence of fossil photosynthetic life and trace the origin of photosynthesis. 2) Studying the mechanism of isotopic fractionation by comparing the Mg isotopic composition measurements with *ab initio* calculations can help us understand the biosynthetic pathway of Mg during chlorophyll formation. In order to understand the origin of these fractionation Black *et al*.^11^ performed *ab initio* calculation to estimate the theoretical isotopic fractionation between chl *a* and chl *b* and predicted an isotopic fractionation of 0.66‰ for the ^26^Mg/^24^Mg ratio (compared to 0.43 ± 0.15 in the natural samples).

Other chlorophyll structures than chl *a* and chl *b* exist, including the important red-shifted chl d and chl *f*. These pigments have not been studied using Mg isotopic methods yet.

Here we report the molecular orbitals of a large variety of Mg-chlorophyll species (chlorophylls a, b, d, f) to obtain the reduced partition function ratio (RPFR) of isotopologues. We then use these data to test whether the Mg isotopic fractionation observed between chl *a* and chl *b* is due to chemical exchange of Mg and predict whether such effect should affect chl *d* and chl *f*. Finally, we propose that by combining our calculations with natural Mg isotopic data would permit to better understand the biosynthetic formation of chlorphyll.

## Methods

Magnesium has only three stable isotopes, ^24^Mg, ^25^Mg and ^26^Mg. The isotopic composition of Mg is usually presented using the δ per mill (‰) notation defined as:

1 $${\delta }^{x}Mg=[\frac{{({}^{x}Mg/{}^{24}Mg)}_{Samples}}{{({}^{x}Mg/{}^{24}Mg)}_{Standard}}-1]\times 1,000$$

with x = 25 or 26. All the data are reported relative to the same standard (DSM-3; Black, 2006 #4698).

And we define the difference of δ^x^Mg between two species X and Y a Δ^x^Mg_X-Y_, with x = 25 or 26 as:

2 $${{\rm{\Delta }}}^{x}Mg(X-Y)={\delta }^{x}Mg(X)-{\delta }^{x}Mg(Y)$$

### Computational method

Orbital geometries, vibrational frequencies, Gibbs free energies of aqueous Mg species are computed using density functional theory (DFT) as implemented by the Gaussian03 code^13^. The DFT method employed is a hybrid density functional consisting of Becke’s three-parameter non-local hybrid exchange potential (B3)^14^ with Lee-Yang-and Parr (LYP) non-local functionals. The 6–311 + G(d,p) basis set, which is an all-electron basis set, will be chosen for H, C, O, and Mg. For the solvation effect, CPCM continuum solvation method (CPCM: conductor-like polarizable continuum model) is used.

**Figure 1:**
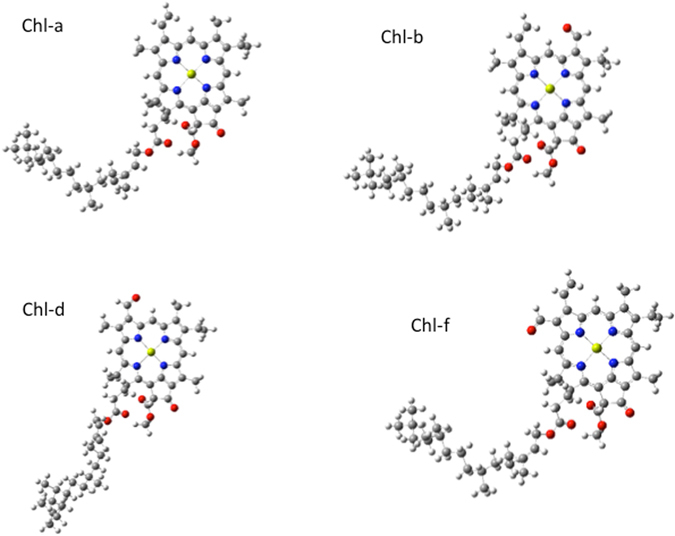
Optimized geometry for chlorophyll (**a**,**b**,**d**, and **f**).

For the structure of chlorophyll a, b, d and f we used an optimized geometry shown in the Fig. 1 (data are available as supplementary materials).

## Results and Discussion

The vibrational modes v_1_, v_2_, and v_3_ of hexaaqua complexes are the fundamental intramolecular vibration modes. The validity of vibrational frequencies and atomic distances of Mg-O of hydrated Mg^2+^ ion computed is discussed in detail in Schott *et al*.^15^. The calculated vibrational frequencies of Mg(H_2_O)_6_^2+^ are underestimated compared with the literature values determined by Raman and IR spectrometry. Setting 12 H_2_O molecules at the second coordination sphere brought the calculation results closer to the literature values. The use of conductor-like polarizable continuum model (CPCM) of solvation for Mg(H_2_O)_6_^2+^ increases vibrational frequencies, but the effect is smaller than setting water molecules in the second coordination sphere.

Calculating how physicochemical properties vary with hydration of the species is a common strategy for examining the accuracy of theoretical calculations of aqueous species^16, 17^. In the theoretical study on the hydration enthalpy of Fe^2+^ and Fe^3+^, Li *et al*.^18^ tested a small cluster model of 6 H_2_O molecules as the first coordination sphere and a large cluster model of 12 additional H_2_O molecules as the second coordination sphere. For Fe^3+^, the large cluster model brought the calculated data closer to the experimental results. For Fe^2+^, both the small and the large cluster modes reproduced the experimental results. Similarly, the hydration enthalpies of Ni^2+^ and Zn^2+^ were appropriately reproduced by using both the small and the large cluster models. Magnesium ion is a divalent one. The small cluster model may be applicable.

The hydration enthalpy of Mg(H_2_O)_*n*_^2+^ (*n* = 6 or 18) was also examined. In order to relate the calculated quantities to the experimental hydration enthalpy (Δ*H*°_hyd_) at 298 K, the following correction terms were considered^18^,

3 $$\begin{array}{rcl}-{\rm{\Delta }}H{^\circ }_{{\rm{hyd}}} & = & -{\rm{\Delta }}{E}_{{\rm{b}}}+{\rm{\Delta }}{E}_{{\rm{sol}}}+n{\rm{\Delta }}{H}_{{\rm{vap}}}+{\rm{\Delta }}nRT-{\rm{\Delta }}E({\rm{Cp}})\\  &  & -{\rm{\Delta }}{E}_{{\rm{zp}}}+{\rm{\Delta }}{E}_{{\rm{rel}}}+{\rm{\Delta }}{E}_{{\rm{geom}}}\end{array}$$

Δ*E*_b_ is the total binding energy of the gas phase cluster [Mg(H_2_O)_*n*_]. Δ*E*_sol_ represents the solvation free energy of Mg(H_2_O)_*n*_^2+^. This term, which contains the entropic contribution to the solvation free energy for the continuum dielectric part, was neglected. Δ*H*_vap_ is the heat of vaporization of water, which is 10.50 kcal/mol^19^. The Δ*E*(Cp) term arises from the difference in heat capacity of the components of the system, which corresponds to a small correction of ~1 kcal/mol at 298 K^18^. Δ*E*_zp_ is the difference in vibrational zero-point energy in forming clusters, Δ*E*_rel_ is a correction due to relativistic effects for metal centers, and Δ*E*_geom_ is a correction due to geometry relaxation for H_2_O during the formation of the clusters. The magnitude of “−Δ*E*_zp_ + Δ*E*_rel_ + Δ*E*_geom_” was reported to be negligibly small (a few kcal/mol)^18^, and hence these corrections were not included in our calculation. The calculated value of Δ*H*°_hyd_ is shown in Table 1. The hydration enthalpy of metal cations has been determined by thermochemical methods^20–22^, and literature values are shown for comparison. Setting 12 H_2_O around the small cluster Mg(H_2_O)_6_^2+^ resulted in the increase of ∆*H* ^0^_hyd_. For the method, O3LYP, large cluster model showed similar value compared with literature data, while B3LYP showed that small cluster model showed similar value compared with literature data. For divalent cations, even small cluster models like Mg(H_2_O)_6_^2+^ can reproduce Δ*H*°_hyd_.

**Table 1:** Hydration enthalpy of Mg(H_2_O)_*n*_^2+^.

Hydration number *n*	Method/Basis set	Δ*H*°_hyd_/kJ/mol	Reference
6	O3LYP/6-31G(d)	−1893	This work
6	B3LYP/6-31G(d)	−1954	This work
18	O3LYP/6-31G(d)	−2047	This work
18	B3LYP/6-31G(d)	−2280	This work
—		−1990	Rosseinsky, 1965
—		−1921	Smith, 1977
—		−1931	Marcus, 1985

**Figure 2:**
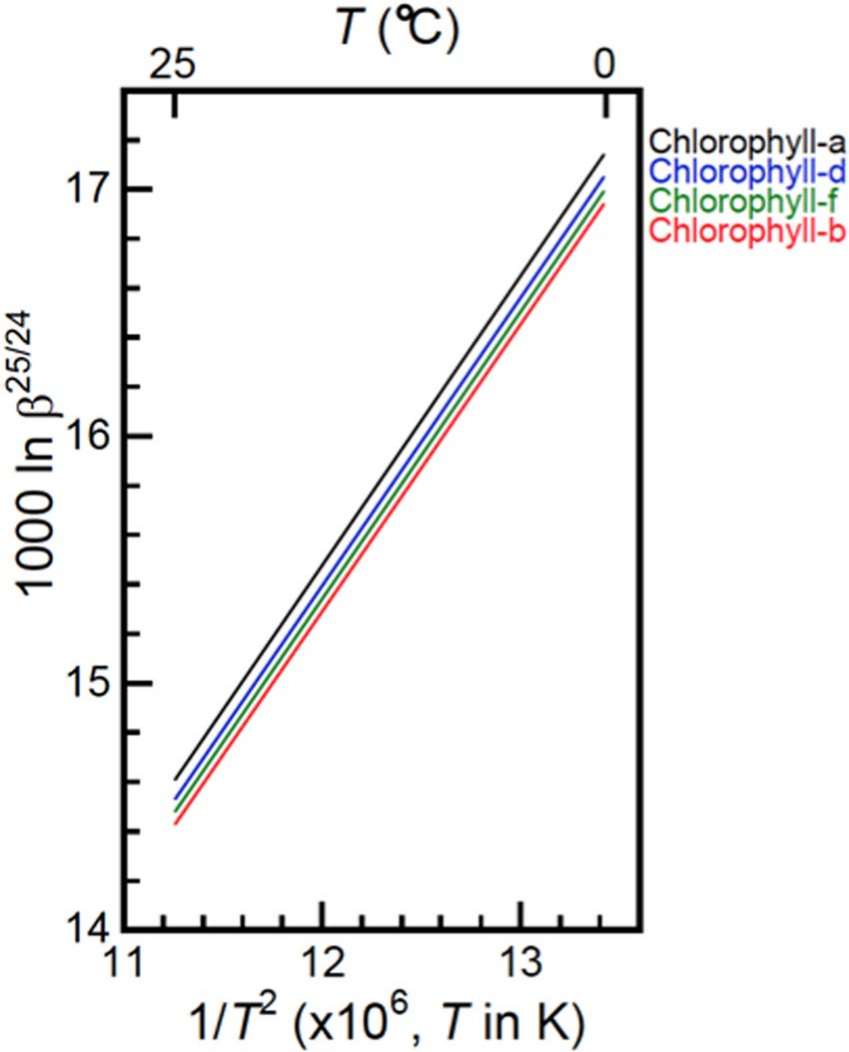
Temperature dependence of ln β. The ln β^25/24^ values of chlorophyll a, b, d, and f calculated by using O3LYP/6-31G(d) are shown as linear functions of *T* ^−2^.

All the data are reported in Table 2 and Fig. 2 for some of the most relevant molecules discussed. The absolute values of our calculated lnβ for Mg(H_2_O)_6_^2+^, Mg(H_2_O)_18_^2+^, chl *a* and chl *b* are in fairly good agreement with literature data^11, 23, 24^ while we are using different program packages: PQS ver. 3.3. for Black *et al*.^11^ vs Gaussian09 in the present study and Schott *et al*.^15^.

**Table 2:** Logarithm of the reduced partition function, ln β (‰), for the pair ^26^Mg-^24^Mg and ^25^Mg-^24^Mg of Mg(II) complexes.

Species	Method/basic sets	lnβ^25/24^ 298 K	lnβ^25/24^ A, B^a^	lnβ^26/24^ 298 K	lnβ^26/24^ A, B^a^	Reference
Mg(H_2_O)_6_^2+^	O3LYP/6-31G(d)	12.43	1.0425, 0.671	23.88	2.0041, 1.272	This study
Mg(H_2_O)_6_^2+^	B3LYP/6-31G(d)	13.73	1.1448, 0.816	26.37	2.1998, 1.556	This study
Mg(H_2_O)_18_^2+^	O3LYP/6-31G(d)	12.48	1.0470, 0.666	23.96	2.0104, 1.282	This study
Mg(H_2_O)_18_^2+^	O3LYP/6-31G(d)	13.18	—	25.22	—	Black *et al*.^11^
Mg(H_2_O)_18_^2+^	B3LYP/6-31G(d)	14.26	1.1881, 0.857	27.40	2.2852, 1.615	This study
Mg(H_2_O)_18_^2+^	BP86/6-31G(d)	—	—	26.74	—	Rustad *et al*.^24^
Chlorophyll-a	O3LYP/6-31G(d)	14.61	1.2077, 0.979	28.07	2.3255, 1.828	This study
Chlorophyll-a	O3LYP/6-31G(d)	15.21		29.27		Black *et al*.^11^
Chlorophyll-a	B3LYP/6-31G(d)	15.14	1.2510, 1.022	29.08	2.4053, 1.944	This study
Chlorophyll-b	O3LYP/6-31G(d)	14.43	1.1949, 0.948	27.73	2.2995, 1.785	This study
Chlorophyll-b	O3LYP/6-31G(d)	14.88	—	28.64	—	Black *et al*.^11^
Chlorophyll-b	B3LYP/6-31G(d)	14.99	1.2397, 1.001	28.79	2.3830, 1.905	
Chlorophyll-d	O3LYP/6-31G(d)	14.53	1.2028, 0.956	27.91	2.3123, 1.822	This study
Chlorophyll-d	B3LYP/6-31G(d)	15.13	1.2509, 1.017	29.08	2.4042, 1.949	This study
Chlorophyll-f	O3LYP/6-31G(d)	14.48	1.1999, 0.937	27.82	2.3063, 1.795	This study
Chlorophyll-f	B3LYP/6-31G(d)	15.03	1.2427, 1.010	28.89	2.3913, 1.906	This study

^**a**^10^3^ ln β = 10^6^ *A* *T* ^−2^ + *B*.

While the relative difference between the lnβ^26/24^Mg of chl *b* and chl *a* (Table 3) that we obtain (0.34) is slightly lower than in Black *et al*.^11^ (0.67); it is closer than the experimental data (0.43). The accuracy of our calculation using Gaussian09 for Mg isotopologues was tested by comparing our calculations with experimental data for a large set of aqueous species (sulphides, citrates, EDTA, oxalates, hydroxides; see ref. 23).

**Table 3:** Theoretical isotopic fractionation between different forms of chlorophylls and chlorophyll *a* for 298 K.

Species	Method	Δ^25^Mg	Δ^26^Mg	Reference
chl *a* vs. chl *b*	O3LYP/6-31G(d)	0.18	0.34	This study
chl *a* vs. chl *b*	O3LYP/6-31G(d)	0.33	0.63	Black *et al*.^11^
chl d vs. chl b	O3LYP/6-31G(d)	0.10	0.18	This study
chl f vs. chl b	O3LYP/6-31G(d)	0.05	0.09	This study

The calculated theoretical isotopic fractionation between the four chlorophylls species considered here (chl *a*, chl *d* and chl *f* compared to chl *b*, see Table 3) predicts resolvable isotopic variations between the different species of chlorophylls. The Mg isotopic composition of the chl *f* should be very close to chl *b* with Δ^26^Mg(chl *f*-chl *b*) = 0.09, chl *d* is slightly heavier, Δ^26^Mg(chl *d*-chl *b*) = 0.18 and finally chl *a* is the isotopically heaviest Δ^26^Mg(chl *a*-chl *b*) = 0.34.

Our results suggest that each type of chlorophyll should exhibit a distinct Mg isotopic composition. It is therefore possible to test whether Mg were exchanged at equilibrium via the Mg isotopic composition of the different chlorophyll. Following this approach, Black *et al*.^11^ proposed that Mg were exchanged at equilibrium between chl *a* and chl *b* based on the similarity between the measured and calculated isotopic composition, suggesting that the timescale of Mg exchanges were similar to the lifetime of the chlorophylls. Our new results show even better agreements with the measurements further suggesting that Mg were exchanged during the lifetime of the chlorophyll.

It is known that the relative abundance of chl *a* and chl *b* in plants or green algae changes as a function of the light conditions^25^ suggesting that the biosynthesis of chl *a* and chl *b* are closely linked. Tanaka *et al*.^26^ isolated the gene encoding for the formation of chl *b* by oxygenation of chl *a* and proposed a chlorophyll cycle with an intermediary molecule, 7-hydroxymethyl. From their model, Mg is not involved in the cycle and, therefore, chl *a* and chl *b* should have similar isotopic composition. The fact that the Mg isotopes are fractionated between isolated chl *a* and chl *b* and follow our theoretical prediction confirms that the biosynthesis of chl *a* and chl *b* are closely linked and implies the Mg is also exchanged during the chlorophyll cycle. Our results imply that the biochemical pathway of the synthesis of chl *a* and chl *b* is more complex that presently modeled.

In this study we have expended our calculations to the two most recently discovered chlorophylls, chl *d* and chl *f*. Both chl *d* and chl *f* are always found in association with chl *a* ^27^. Chlorphyll *d* and *f* are red-shifted chlorophyll that can adsorb light with wavelength up to 760 nm while chl *a* and *b* do not absorb light <700 nm. These pigments have only been discovered in a limited set of organisms so far (e.g. *Acaryochloris Marina*, *Halomicronema Hongdechloris*) and are supposed to represent the consequence of adaptation of the micro-organisms to specific ecological niches. The understanding of the biosynthesis of these novel pigments is still limited and while there is no consensus on the existence of chl *d*-chl *a* and/or chl *f*-chl *a* cycles there are hints that the biosynthesis of chl *d* and *f* are linked to ch*l a*. Using spiked oxygen marking Schliep *et al*.^28^ suggested that chl d was formed directly from chl *a* via oxygenase-type reactions but the enzymes responsible for the synthesis have yet to be discovered. Our new results on the two long wave-length absorbing chlorophylls exhibit distinct isotopic composition suggesting that it would also be possible to test the kinetic of Mg exchanged compared to the lifetime of chl *d* and chl *f*.

Isotopic fractionation of Mg at equilibrium between 4 types of chlorophylls (*a, b, d*, and *f*) was demonstrated theoretically. We show that our data are consistent with previous calculations and confirms that chl *a* and chl *b* are isotopically fractionated in plants following a thermodynamic equilibrium. This implies that during the synthesis of chl *b* and chl *a*, Mg is also exchanged. We expand these calculations to the two latest discovered pigments, chl *d* and chl *f* and show that their Mg isotopes should also be isotopically fractionated during equilibrium exchanges reactions.

## Electronic supplementary material

Supplementary Information
