# Supplementary material for: Theoretical isotopic fractionation of magnesium between chlorophylls
Source: Sci Rep. 2017 Aug 1;7:6973. doi: 10.1038/s41598-017-07305-6 (PMC5539320; doi:10.1038/s41598-017-07305-6)
Supplement: Supplementary file 1 — Supplementary Information [file 41598_2017_7305_MOESM1_ESM.doc]

**Supporting Information**

# Theoretical isotopic fractionation of magnesium between chlorophyll

# Frédéric Moynier^1,2^ and Toshiyuki Fujii^3^

^1^ Institut de Physique du Globe de Paris, Sorbonne Paris Cité, Université Paris Diderot, CNRS, F-75005 Paris, France

^2^ Institut Universitaire de France, Paris, France

^3^ Division of Sustainable Energy and Environmental Engineering, Graduate School of Engineering, Osaka University, 2-1 Yamadaoka, Suita, Osaka 565-0871, Japan

# Table S1 Optimized structure Cartesian coordinates of chlorophyll a, b, d, and f (Method/Basis set: O3LYP/6-31G(d))

Chlorophyll-a

| Mg | 0.112192 | 0.611338 | -1.702022 |
| --- | --- | --- | --- |
| O | -1.325528 | 0.105164 | 5.356894 |
| O | -2.869986 | 0.979931 | 6.648611 |
| O | -0.513997 | -2.524719 | 2.812280 |
| O | 0.836546 | -2.371125 | 4.626853 |
| O | 3.460760 | -1.433173 | 3.470927 |
| N | 1.680104 | -0.029879 | -0.618109 |
| N | 1.362952 | 0.599575 | -3.350921 |
| N | -1.455850 | 1.231613 | -2.839262 |
| N | -1.102690 | 0.581602 | 0.064134 |
| C | -2.428677 | 3.194097 | -6.764885 |
| C | 1.596499 | -0.307314 | 0.684911 |
| C | 2.831867 | -0.798281 | 1.218402 |
| C | 3.743601 | -0.827445 | 0.194839 |
| C | 2.977351 | -0.336979 | -0.992122 |
| C | 3.431319 | -0.208748 | -2.292908 |
| C | 2.661573 | 0.228554 | -3.415606 |
| C | -1.428295 | 1.528703 | -4.151982 |
| C | -0.286719 | 1.363075 | -4.992350 |
| C | 0.983351 | 0.937957 | -4.636481 |
| C | 2.122270 | 0.773122 | -5.555869 |
| C | 2.073679 | 1.046863 | -7.022735 |
| C | 3.171774 | 0.332120 | -4.794689 |
| C | 4.562297 | -0.021600 | -5.233298 |
| C | 4.763566 | -1.524389 | -5.506595 |
| C | -2.766431 | 1.965600 | -4.609272 |
| C | -3.598261 | 1.854431 | -3.520603 |
| C | -2.760415 | 1.413829 | -2.402391 |
| C | -3.181503 | 1.204138 | -1.103202 |
| C | -2.417221 | 0.808945 | 0.043666 |
| C | -0.711947 | 0.255214 | 1.366996 |
| C | -1.845039 | 0.491816 | 2.332064 |
| C | -3.066130 | 0.568627 | 1.387363 |
| C | -3.915918 | -0.723147 | 1.380047 |
| C | -1.615629 | 1.792384 | 3.157560 |
| C | -2.529015 | 1.943765 | 4.381043 |
| C | -2.192302 | 0.945111 | 5.488548 |
| C | -3.901242 | 1.941697 | 6.985120 |
| C | -3.703916 | 2.328646 | 8.415159 |
| C | -4.606629 | 2.276619 | 9.429001 |
| C | -6.018393 | 1.760430 | 9.296462 |
| C | -6.144609 | 0.268823 | 9.697678 |
| C | -7.587069 | -0.269900 | 9.669215 |
| C | -8.573525 | 0.289066 | 10.714831 |
| C | -8.115070 | 0.016298 | 12.155344 |
| C | -10.003035 | -0.274325 | 10.510914 |
| C | -10.718397 | 0.156630 | 9.223366 |
| C | -12.192946 | -0.285078 | 9.210260 |
| C | -12.949141 | -0.009205 | 7.893179 |
| C | -13.033193 | 1.486581 | 7.561933 |
| C | -14.379802 | -0.618032 | 7.924774 |
| C | -14.458014 | -2.147137 | 7.945194 |
| C | -15.905523 | -2.702502 | 7.836532 |
| C | -16.321466 | -3.220845 | 6.441039 |
| C | -16.311579 | -2.148745 | 5.347437 |
| C | -17.704735 | -3.888864 | 6.533714 |
| C | -4.204854 | 2.752700 | 10.797239 |
| C | 0.564827 | -0.202089 | 1.648491 |
| C | 1.128275 | -0.736100 | 2.960724 |
| C | 0.374622 | -1.990845 | 3.445853 |
| C | 0.203729 | -3.514871 | 5.235930 |
| C | 2.646477 | -1.060513 | 2.662679 |
| C | -5.057093 | 2.157328 | -3.429379 |
| C | -3.142416 | 2.394963 | -5.954284 |
| C | 5.168864 | -1.249187 | 0.215917 |
| H | -4.237988 | 1.356531 | -0.911503 |
| H | -0.449607 | 1.575447 | -6.041138 |
| H | 4.463291 | -0.475200 | -2.487225 |
| H | 5.341612 | -2.092798 | -0.464366 |
| H | 5.827679 | -0.434702 | -0.110393 |
| H | 5.464483 | -1.551257 | 1.221884 |
| H | 5.283901 | 0.314588 | -4.479088 |
| H | 4.805138 | 0.541843 | -6.139236 |
| H | 4.100185 | -1.872514 | -6.303999 |
| H | 5.795336 | -1.710585 | -5.819275 |
| H | 4.565603 | -2.133592 | -4.618488 |
| H | 1.756648 | 2.076193 | -7.227965 |
| H | 3.047750 | 0.902183 | -7.492304 |
| H | 1.363294 | 0.380963 | -7.528072 |
| H | -4.122772 | 2.065422 | -6.293977 |
| H | -5.250230 | 2.963992 | -2.711613 |
| H | -5.462187 | 2.476150 | -4.390722 |
| H | -5.628084 | 1.281627 | -3.098192 |
| H | 1.099704 | -0.015496 | 3.784444 |
| H | 0.248907 | -4.378039 | 4.568467 |
| H | 0.771498 | -3.699481 | 6.146949 |
| H | -0.836042 | -3.276853 | 5.471604 |
| H | -3.723881 | 1.411763 | 1.630611 |
| H | -4.379489 | -0.858601 | 2.361347 |
| H | -4.714887 | -0.676534 | 0.634388 |
| H | -3.295842 | -1.601011 | 1.175886 |
| H | -1.915823 | -0.346781 | 3.025230 |
| H | -1.753997 | 2.660464 | 2.501721 |
| H | -0.579308 | 1.822746 | 3.501968 |
| H | -3.588762 | 1.850386 | 4.111456 |
| H | -2.412027 | 2.964208 | 4.766401 |
| H | -4.867471 | 1.468452 | 6.794634 |
| H | -3.820370 | 2.828317 | 6.345339 |
| H | -2.704469 | 2.702433 | 8.632222 |
| H | -4.793398 | 3.635834 | 11.080910 |
| H | -3.145719 | 3.013992 | 10.857146 |
| H | -6.667638 | 2.363376 | 9.940548 |
| H | -6.398943 | 1.884370 | 8.276939 |
| H | -4.414199 | 1.990639 | 11.557772 |
| H | -5.702581 | 0.115827 | 10.687252 |
| H | -5.536427 | -0.327358 | 9.005838 |
| H | -7.991993 | -0.108832 | 8.662450 |
| H | -7.529680 | -1.359875 | 9.799531 |
| H | -8.645798 | 1.379053 | 10.585376 |
| H | -9.967185 | -1.371382 | 10.565886 |
| H | -10.610036 | 0.048974 | 11.366363 |
| H | -10.214277 | -0.264325 | 8.343802 |
| H | -10.654806 | 1.247642 | 9.127842 |
| H | -12.227882 | -1.359388 | 9.424288 |
| H | -12.723435 | 0.207955 | 10.036685 |
| H | -12.400622 | -0.505359 | 7.077815 |
| H | -14.923108 | -0.215059 | 8.790402 |
| H | -14.906245 | -0.240058 | 7.039550 |
| H | -14.024293 | -2.526203 | 8.877506 |
| H | -13.839976 | -2.561474 | 7.137406 |
| H | -16.011893 | -3.532583 | 8.545019 |
| H | -16.624340 | -1.939418 | 8.164346 |
| H | -15.595555 | -3.996781 | 6.155385 |
| H | -18.478394 | -3.164701 | 6.815103 |
| H | -17.988757 | -4.320769 | 5.567029 |
| H | -7.151497 | 0.484385 | 12.382035 |
| H | -8.839785 | 0.410380 | 12.875346 |
| H | -8.013359 | -1.059291 | 12.343835 |
| H | -12.042030 | 1.936821 | 7.443270 |
| H | -13.576678 | 1.651504 | 6.624862 |
| H | -13.558137 | 2.039289 | 8.350420 |
| H | -15.314545 | -1.718782 | 5.202303 |
| H | -16.621180 | -2.572960 | 4.385139 |
| H | -17.003267 | -1.330926 | 5.583504 |
| H | -17.711691 | -4.693212 | 7.277016 |
| H | -1.472873 | 3.625192 | -6.479445 |
| H | -2.815208 | 3.479043 | -7.738856 |

Chlorophyll-b

| Mg | 0.143534 | 0.593574 | -1.716210 |
| --- | --- | --- | --- |
| O | -1.331481 | 0.082330 | 5.334003 |
| O | -2.888134 | 0.951285 | 6.614638 |
| O | -0.485925 | -2.534949 | 2.785497 |
| O | 0.850225 | -2.382664 | 4.610803 |
| O | 3.478504 | -1.418784 | 3.479279 |
| O | 1.228896 | 1.476378 | -7.625489 |
| N | 1.715596 | -0.030509 | -0.625015 |
| N | 1.413091 | 0.590201 | -3.366355 |
| N | -1.420643 | 1.207414 | -2.862463 |
| N | -1.077101 | 0.559609 | 0.042979 |
| C | -2.381187 | 3.054832 | -6.857295 |
| C | 1.623746 | -0.307083 | 0.681745 |
| C | 2.859390 | -0.788822 | 1.222547 |
| C | 3.777523 | -0.812585 | 0.205003 |
| C | 3.015128 | -0.329086 | -0.986413 |
| C | 3.479601 | -0.198050 | -2.286107 |
| C | 2.715701 | 0.232604 | -3.409299 |
| C | -1.388832 | 1.499657 | -4.175662 |
| C | -0.245930 | 1.339972 | -5.011749 |
| C | 1.030004 | 0.932063 | -4.648488 |
| C | 2.179221 | 0.786988 | -5.550003 |
| C | 2.192650 | 1.074324 | -7.000177 |
| C | 3.237403 | 0.350548 | -4.787711 |
| C | 4.633019 | 0.010358 | -5.213036 |
| C | 4.835152 | -1.493535 | -5.494115 |
| C | -2.731615 | 1.929770 | -4.639339 |
| C | -3.564099 | 1.819274 | -3.549028 |
| C | -2.729753 | 1.387118 | -2.431504 |
| C | -3.154275 | 1.177490 | -1.130742 |
| C | -2.396081 | 0.785904 | 0.015076 |
| C | -0.693475 | 0.240028 | 1.344110 |
| C | -1.830207 | 0.470681 | 2.305517 |
| C | -3.047905 | 0.543155 | 1.356763 |
| C | -3.892542 | -0.752174 | 1.347755 |
| C | -1.609042 | 1.771568 | 3.133509 |
| C | -2.531493 | 1.919383 | 4.350755 |
| C | -2.200931 | 0.920171 | 5.459353 |
| C | -3.922740 | 1.910076 | 6.946127 |
| C | -3.724784 | 2.307389 | 8.373275 |
| C | -4.628476 | 2.265906 | 9.387944 |
| C | -6.040951 | 1.751991 | 9.258661 |
| C | -6.166426 | 0.260499 | 9.662321 |
| C | -7.610408 | -0.275370 | 9.646988 |
| C | -8.585211 | 0.283469 | 10.703204 |
| C | -8.112305 | 0.010358 | 12.138964 |
| C | -10.017281 | -0.281121 | 10.515011 |
| C | -10.745624 | 0.150393 | 9.235035 |
| C | -12.221343 | -0.289596 | 9.241498 |
| C | -12.993304 | -0.011755 | 7.933745 |
| C | -13.077589 | 1.483365 | 7.601581 |
| C | -14.427180 | -0.616000 | 7.989605 |
| C | -14.509611 | -2.144036 | 8.004447 |
| C | -15.962763 | -2.692809 | 7.925638 |
| C | -16.411997 | -3.195676 | 6.534349 |
| C | -16.425318 | -2.113439 | 5.451507 |
| C | -17.796156 | -3.859039 | 6.656592 |
| C | -4.225446 | 2.751281 | 10.752106 |
| C | 0.588641 | -0.208799 | 1.635768 |
| C | 1.144640 | -0.738926 | 2.953065 |
| C | 0.395308 | -1.999200 | 3.428241 |
| C | 0.219643 | -3.533192 | 5.209516 |
| C | 2.666859 | -1.052137 | 2.665701 |
| C | -5.024915 | 2.116358 | -3.456397 |
| C | -3.116565 | 2.345338 | -5.981684 |
| C | 5.205849 | -1.223934 | 0.235068 |
| H | -4.212113 | 1.326287 | -0.944235 |
| H | -0.381212 | 1.539452 | -6.065648 |
| H | 4.515344 | -0.455824 | -2.471181 |
| H | 5.389652 | -2.065714 | -0.444573 |
| H | 5.861056 | -0.404133 | -0.085258 |
| H | 5.496853 | -1.524868 | 1.242732 |
| H | 5.338786 | 0.336312 | -4.440175 |
| H | 4.893566 | 0.579481 | -6.109443 |
| H | 4.188680 | -1.831976 | -6.309037 |
| H | 5.873673 | -1.673755 | -5.786393 |
| H | 4.618684 | -2.108865 | -4.615207 |
| H | 3.159250 | 0.902165 | -7.509675 |
| H | -4.133697 | 2.087119 | -6.269703 |
| H | -5.220357 | 2.925583 | -2.742253 |
| H | -5.434549 | 2.429284 | -4.417557 |
| H | -5.591627 | 1.239403 | -3.121439 |
| H | 1.105038 | -0.019277 | 3.777121 |
| H | 0.274634 | -4.392610 | 4.537978 |
| H | 0.781921 | -3.719209 | 6.123642 |
| H | -0.823191 | -3.302465 | 5.438837 |
| H | -3.709807 | 1.383562 | 1.597700 |
| H | -4.357598 | -0.889711 | 2.328084 |
| H | -4.689691 | -0.707941 | 0.600140 |
| H | -3.268647 | -1.627512 | 1.144450 |
| H | -1.898764 | -0.368714 | 2.998042 |
| H | -1.745865 | 2.639402 | 2.477133 |
| H | -0.575197 | 1.805007 | 3.484983 |
| H | -3.588954 | 1.824145 | 4.073059 |
| H | -2.419214 | 2.939690 | 4.738028 |
| H | -4.887046 | 1.430927 | 6.760502 |
| H | -3.848064 | 2.793273 | 6.300707 |
| H | -2.724336 | 2.679762 | 8.588062 |
| H | -4.809845 | 3.639892 | 11.027397 |
| H | -3.165069 | 3.007449 | 10.811356 |
| H | -6.688134 | 2.355926 | 9.903771 |
| H | -6.423380 | 1.874669 | 8.239700 |
| H | -4.440414 | 1.997031 | 11.518909 |
| H | -5.715797 | 0.107564 | 10.647998 |
| H | -5.565621 | -0.337525 | 8.965719 |
| H | -8.024771 | -0.112217 | 8.644401 |
| H | -7.552959 | -1.365575 | 9.775275 |
| H | -8.660045 | 1.373320 | 10.574534 |
| H | -9.980092 | -1.378054 | 10.569634 |
| H | -10.614580 | 0.042424 | 11.377038 |
| H | -10.252599 | -0.272107 | 8.350041 |
| H | -10.682301 | 1.241192 | 9.138191 |
| H | -12.255325 | -1.363827 | 9.455630 |
| H | -12.740001 | 0.204751 | 10.074451 |
| H | -12.459483 | -0.511976 | 7.111427 |
| H | -14.953134 | -0.214169 | 8.866168 |
| H | -14.965728 | -0.230926 | 7.114663 |
| H | -14.059328 | -2.530398 | 8.925950 |
| H | -13.911029 | -2.557270 | 7.181854 |
| H | -16.054395 | -3.528944 | 8.628869 |
| H | -16.670681 | -1.930256 | 8.276944 |
| H | -15.696421 | -3.971667 | 6.224458 |
| H | -18.559741 | -3.133875 | 6.961464 |
| H | -18.103609 | -4.282099 | 5.693101 |
| H | -7.147266 | 0.480015 | 12.356162 |
| H | -8.830227 | 0.403120 | 12.866398 |
| H | -8.006893 | -1.065104 | 12.325886 |
| H | -12.086377 | 1.929369 | 7.467876 |
| H | -13.633153 | 1.648902 | 6.671791 |
| H | -13.589048 | 2.039316 | 8.396539 |
| H | -15.429952 | -1.687370 | 5.284428 |
| H | -16.760985 | -2.527019 | 4.493313 |
| H | -17.106887 | -1.294880 | 5.712720 |
| H | -17.787996 | -4.668996 | 7.393571 |
| H | -1.379238 | 3.416191 | -6.647716 |
| H | -2.794974 | 3.326943 | -7.823599 |

Chlorophyll-d

| Mg | 0.791695 | 0.788996 | -1.842661 |
| --- | --- | --- | --- |
| O | -3.465331 | 0.166189 | 3.983196 |
| O | -5.020849 | 1.447747 | 4.851241 |
| O | -2.562596 | -2.291405 | 1.271611 |
| O | -2.090960 | -2.875394 | 3.410775 |
| O | 0.926385 | -2.907523 | 3.489193 |
| O | 1.298552 | 3.819781 | -7.032024 |
| N | 1.492897 | -0.519239 | -0.487703 |
| N | 2.521634 | 0.459576 | -2.926588 |
| N | 0.088667 | 2.075251 | -3.259723 |
| N | -0.966149 | 1.077214 | -0.637028 |
| C | 0.811906 | -0.871609 | 0.600367 |
| C | 1.497491 | -1.855264 | 1.382103 |
| C | 2.683470 | -2.133728 | 0.750333 |
| C | 2.662947 | -1.266655 | -0.466051 |
| C | 3.619227 | -1.182403 | -1.458409 |
| C | 3.554992 | -0.358462 | -2.630882 |
| C | 0.727110 | 2.439807 | -4.389379 |
| C | 2.017578 | 1.972082 | -4.787827 |
| C | 2.834125 | 1.069812 | -4.129440 |
| C | 4.149143 | 0.605078 | -4.601169 |
| C | 4.800355 | 1.056895 | -5.865509 |
| C | 4.599422 | -0.289869 | -3.665664 |
| C | 5.872438 | -1.084221 | -3.668687 |
| C | 5.715420 | -2.506720 | -4.238919 |
| C | -0.105364 | 3.380471 | -5.154357 |
| C | -1.268791 | 3.557967 | -4.442577 |
| C | -1.133397 | 2.721237 | -3.235668 |
| C | -2.073646 | 2.587258 | -2.234590 |
| C | -2.006533 | 1.808679 | -1.027347 |
| C | -1.254818 | 0.490841 | 0.604108 |
| C | -2.530987 | 1.060593 | 1.170733 |
| C | -3.172848 | 1.722656 | -0.070556 |
| C | -4.336590 | 0.900528 | -0.670590 |
| C | -2.246772 | 2.057785 | 2.331018 |
| C | -3.478711 | 2.428424 | 3.166084 |
| C | -3.976750 | 1.268010 | 4.030394 |
| C | -5.671674 | 2.731313 | 5.110358 |
| C | -5.092401 | 3.406919 | 6.311599 |
| C | -5.575175 | 3.366515 | 7.580601 |
| C | -6.799602 | 2.594152 | 8.006587 |
| C | -6.454310 | 1.160296 | 8.481126 |
| C | -7.667960 | 0.353331 | 8.978943 |
| C | -8.352335 | 0.824000 | 10.278164 |
| C | -7.395668 | 0.817387 | 11.479904 |
| C | -9.599683 | -0.036961 | 10.611600 |
| C | -10.785288 | 0.097979 | 9.646542 |
| C | -12.026075 | -0.653107 | 10.167544 |
| C | -13.230904 | -0.662810 | 9.201946 |
| C | -13.794029 | 0.738194 | 8.939176 |
| C | -14.365242 | -1.589218 | 9.742194 |
| C | -14.064589 | -3.088387 | 9.718218 |
| C | -15.272287 | -3.969263 | 10.156100 |
| C | -16.048821 | -4.644717 | 9.002578 |
| C | -16.728215 | -3.661329 | 8.046569 |
| C | -17.078782 | -5.626080 | 9.594969 |
| C | -4.862571 | 4.116341 | 8.671771 |
| C | -0.427989 | -0.463384 | 1.162032 |
| C | -0.645900 | -1.312214 | 2.408442 |
| C | -1.888122 | -2.218557 | 2.278883 |
| C | -3.240562 | -3.744589 | 3.459841 |
| C | 0.673222 | -2.168661 | 2.569770 |
| C | -2.460081 | 4.397829 | -4.758185 |
| C | 0.250042 | 4.010140 | -6.445411 |
| C | 3.761966 | -3.076011 | 1.148804 |
| H | -2.996823 | 3.141669 | -2.363353 |
| H | 2.371510 | 2.380233 | -5.726430 |
| H | 4.502703 | -1.800478 | -1.351483 |
| H | 3.917305 | -3.846311 | 0.382809 |
| H | 4.718170 | -2.552834 | 1.274870 |
| H | 3.511731 | -3.570037 | 2.088963 |
| H | 6.276946 | -1.138608 | -2.650759 |
| H | 6.625993 | -0.546956 | -4.252241 |
| H | 5.379998 | -2.479594 | -5.279997 |
| H | 6.676415 | -3.028651 | -4.206696 |
| H | 4.990827 | -3.099500 | -3.670688 |
| H | 4.881636 | 2.149184 | -5.905775 |
| H | 5.804477 | 0.643866 | -5.972572 |
| H | 4.219591 | 0.746034 | -6.742806 |
| H | -0.512839 | 4.690086 | -6.868278 |
| H | -2.646980 | 5.127743 | -3.961406 |
| H | -2.345287 | 4.949573 | -5.691140 |
| H | -3.360748 | 3.778299 | -4.848370 |
| H | -0.780152 | -0.730724 | 3.326482 |
| H | -3.199776 | -4.478659 | 2.652291 |
| H | -3.182869 | -4.233891 | 4.431287 |
| H | -4.154129 | -3.150896 | 3.380599 |
| H | -3.541579 | 2.732619 | 0.146790 |
| H | -5.163368 | 0.865742 | 0.044607 |
| H | -4.713667 | 1.345489 | -1.596195 |
| H | -4.026485 | -0.128113 | -0.877188 |
| H | -3.146841 | 0.250346 | 1.562816 |
| H | -1.810039 | 2.974872 | 1.917309 |
| H | -1.497299 | 1.627378 | 2.999383 |
| H | -4.297956 | 2.788034 | 2.530706 |
| H | -3.217045 | 3.272733 | 3.814273 |
| H | -6.717603 | 2.458056 | 5.248622 |
| H | -5.606727 | 3.370010 | 4.225457 |
| H | -4.181911 | 3.978943 | 6.137057 |
| H | -5.506577 | 4.913313 | 9.067029 |
| H | -3.927899 | 4.569597 | 8.332135 |
| H | -7.289207 | 3.140473 | 8.819842 |
| H | -7.532399 | 2.526067 | 7.195624 |
| H | -4.638142 | 3.457164 | 9.519368 |
| H | -5.683150 | 1.205808 | 9.257350 |
| H | -6.001720 | 0.621806 | 7.640042 |
| H | -8.407292 | 0.318674 | 8.169462 |
| H | -7.331315 | -0.683002 | 9.123937 |
| H | -8.710359 | 1.854307 | 10.137540 |
| H | -9.297439 | -1.091123 | 10.683149 |
| H | -9.939222 | 0.247963 | 11.615619 |
| H | -10.523497 | -0.292696 | 8.655177 |
| H | -11.021573 | 1.160580 | 9.513265 |
| H | -11.731193 | -1.685475 | 10.386374 |
| H | -12.338520 | -0.213587 | 11.124595 |
| H | -12.896534 | -1.081798 | 8.241233 |
| H | -14.624764 | -1.282574 | 10.764124 |
| H | -15.253228 | -1.385232 | 9.130646 |
| H | -13.229827 | -3.313569 | 10.391696 |
| H | -13.731329 | -3.384834 | 8.715210 |
| H | -14.899742 | -4.756959 | 10.821364 |
| H | -15.971914 | -3.375375 | 10.759095 |
| H | -15.322279 | -5.232942 | 8.423030 |
| H | -17.833661 | -5.100806 | 10.191220 |
| H | -17.599399 | -6.159741 | 8.791189 |
| H | -6.540389 | 1.485029 | 11.331935 |
| H | -7.907997 | 1.149365 | 12.388822 |
| H | -7.002367 | -0.188558 | 11.669711 |
| H | -13.045193 | 1.400473 | 8.491845 |
| H | -14.641345 | 0.698633 | 8.245900 |
| H | -14.143861 | 1.207594 | 9.866403 |
| H | -16.005694 | -3.003639 | 7.550538 |
| H | -17.266545 | -4.196005 | 7.255509 |
| H | -17.456451 | -3.032682 | 8.572991 |
| H | -16.597823 | -6.370107 | 10.238390 |

Chlorophyll-f

| Mg | 0.096879 | 0.587452 | -1.678750 |
| --- | --- | --- | --- |
| O | -1.196785 | 0.109917 | 5.423901 |
| O | -2.753866 | 0.954095 | 6.721062 |
| O | -0.330841 | -2.515646 | 2.872230 |
| O | 1.039024 | -2.298508 | 4.665806 |
| O | 3.617300 | -1.303554 | 3.440474 |
| O | -5.785110 | 1.891798 | -2.322901 |
| N | 1.707500 | 0.006427 | -0.622323 |
| N | 1.307997 | 0.588420 | -3.357219 |
| N | -1.520073 | 1.155811 | -2.794975 |
| N | -1.072500 | 0.546120 | 0.115056 |
| C | -2.605592 | 3.036225 | -6.716984 |
| C | 1.660802 | -0.257990 | 0.687019 |
| C | 2.921080 | -0.709786 | 1.196299 |
| C | 3.808973 | -0.726554 | 0.151913 |
| C | 3.001736 | -0.271094 | -1.021747 |
| C | 3.422381 | -0.149004 | -2.336280 |
| C | 2.617476 | 0.249967 | -3.444342 |
| C | -1.518582 | 1.435754 | -4.111875 |
| C | -0.394631 | 1.284669 | -4.973428 |
| C | 0.894499 | 0.896266 | -4.637464 |
| C | 2.016303 | 0.746950 | -5.579618 |
| C | 1.928382 | 0.995979 | -7.049150 |
| C | 3.093606 | 0.345471 | -4.835654 |
| C | 4.482302 | 0.019860 | -5.301025 |
| C | 4.713821 | -1.481254 | -5.559383 |
| C | -2.879898 | 1.834371 | -4.544753 |
| C | -3.679241 | 1.714683 | -3.423639 |
| C | -2.813185 | 1.308572 | -2.319791 |
| C | -3.197662 | 1.102746 | -1.007066 |
| C | -2.394705 | 0.739464 | 0.120613 |
| C | -0.647133 | 0.245859 | 1.411045 |
| C | -1.766055 | 0.458548 | 2.397628 |
| C | -3.007927 | 0.498346 | 1.478845 |
| C | -3.831256 | -0.810757 | 1.500413 |
| C | -1.554396 | 1.770293 | 3.210672 |
| C | -2.456391 | 1.909275 | 4.443957 |
| C | -2.084010 | 0.927704 | 5.554330 |
| C | -3.810904 | 1.886400 | 7.051532 |
| C | -3.647876 | 2.249468 | 8.492229 |
| C | -4.580762 | 2.204787 | 9.480781 |
| C | -5.996860 | 1.717344 | 9.302700 |
| C | -6.160347 | 0.223696 | 9.685297 |
| C | -7.613652 | -0.283674 | 9.627764 |
| C | -8.603573 | 0.283972 | 10.664850 |
| C | -8.164639 | 0.002874 | 12.109784 |
| C | -10.037995 | -0.264524 | 10.446832 |
| C | -10.736167 | 0.171119 | 9.151496 |
| C | -12.215933 | -0.255429 | 9.131591 |
| C | -12.958848 | 0.023825 | 7.807522 |
| C | -13.030268 | 1.518464 | 7.471232 |
| C | -14.396900 | -0.574252 | 7.835486 |
| C | -14.485711 | -2.101682 | 7.847512 |
| C | -15.940526 | -2.643331 | 7.745699 |
| C | -16.368056 | -3.146469 | 6.347736 |
| C | -16.359658 | -2.065705 | 5.263526 |
| C | -17.756625 | -3.804680 | 6.448120 |
| C | -4.207393 | 2.659384 | 10.863758 |
| C | 0.650098 | -0.171218 | 1.670886 |
| C | 1.255914 | -0.675634 | 2.976028 |
| C | 0.549025 | -1.946639 | 3.487176 |
| C | 0.451398 | -3.455151 | 5.295617 |
| C | 2.775515 | -0.961547 | 2.646994 |
| C | -5.124933 | 2.004698 | -3.339738 |
| C | -3.295280 | 2.237448 | -5.882425 |
| C | 5.245175 | -1.110061 | 0.143440 |
| H | -4.254797 | 1.238759 | -0.811702 |
| H | -0.583440 | 1.472051 | -6.022371 |
| H | 4.456479 | -0.390695 | -2.550308 |
| H | 5.424066 | -1.957995 | -0.529861 |
| H | 5.873815 | -0.282846 | -0.209279 |
| H | 5.572934 | -1.390684 | 1.145660 |
| H | 5.212242 | 0.383409 | -4.567952 |
| H | 4.691095 | 0.576532 | -6.219551 |
| H | 4.042534 | -1.855585 | -6.338089 |
| H | 5.743012 | -1.647108 | -5.891407 |
| H | 4.548918 | -2.082936 | -4.659522 |
| H | 1.569468 | 2.009567 | -7.263502 |
| H | 2.897781 | 0.880764 | -7.536306 |
| H | 1.234236 | 0.295917 | -7.530498 |
| H | -4.281958 | 1.902130 | -6.194831 |
| H | -5.595647 | 2.346248 | -4.280771 |
| H | 1.225990 | 0.052225 | 3.793218 |
| H | 0.514263 | -4.323418 | 4.636267 |
| H | 1.037654 | -3.612201 | 6.200067 |
| H | -0.591913 | -3.247391 | 5.543884 |
| H | -3.680142 | 1.328859 | 1.725336 |
| H | -4.267672 | -0.948178 | 2.493858 |
| H | -4.648413 | -0.782413 | 0.774161 |
| H | -3.198504 | -1.677300 | 1.286622 |
| H | -1.799958 | -0.377041 | 3.097313 |
| H | -1.722237 | 2.629726 | 2.550567 |
| H | -0.514799 | 1.827181 | 3.541618 |
| H | -3.516718 | 1.790225 | 4.187402 |
| H | -2.358270 | 2.934937 | 4.820948 |
| H | -4.765639 | 1.401846 | 6.832036 |
| H | -3.733549 | 2.788493 | 6.432345 |
| H | -2.648217 | 2.601885 | 8.741327 |
| H | -4.786434 | 3.551320 | 11.139757 |
| H | -3.145525 | 2.899083 | 10.955446 |
| H | -6.653033 | 2.325023 | 9.935191 |
| H | -6.346069 | 1.857673 | 8.274201 |
| H | -4.452984 | 1.894954 | 11.611122 |
| H | -5.738341 | 0.051076 | 10.680155 |
| H | -5.553640 | -0.377872 | 8.996953 |
| H | -7.999703 | -0.102917 | 8.617064 |
| H | -7.579796 | -1.375969 | 9.746648 |
| H | -8.663762 | 1.374814 | 10.536672 |
| H | -10.014045 | -1.361687 | 10.504289 |
| H | -10.649285 | 0.067324 | 11.295823 |
| H | -10.230600 | -0.259028 | 8.277435 |
| H | -10.660965 | 1.260969 | 9.052589 |
| H | -12.263717 | -1.328317 | 9.349603 |
| H | -12.746144 | 0.247391 | 9.952048 |
| H | -12.410762 | -0.480329 | 6.997191 |
| H | -14.937751 | -0.170407 | 8.701955 |
| H | -14.916440 | -0.186434 | 6.950306 |
| H | -14.051803 | -2.491465 | 8.775365 |
| H | -13.876585 | -2.516531 | 7.033613 |
| H | -16.048016 | -3.477401 | 8.449098 |
| H | -16.650416 | -1.876456 | 8.083305 |
| H | -15.650292 | -3.925514 | 6.050964 |
| H | -18.522447 | -3.076470 | 6.739767 |
| H | -18.049536 | -4.227360 | 5.479942 |
| H | -7.200992 | 0.464353 | 12.349360 |
| H | -8.895144 | 0.399487 | 12.822384 |
| H | -8.071940 | -1.073897 | 12.295877 |
| H | -12.034678 | 1.960639 | 7.358845 |
| H | -13.563857 | 1.684454 | 6.528768 |
| H | -13.557452 | 2.077592 | 8.253563 |
| H | -15.360337 | -1.642685 | 5.112923 |
| H | -16.679778 | -2.479474 | 4.300137 |
| H | -17.043283 | -1.244859 | 5.511892 |
| H | -17.763402 | -4.614038 | 7.185760 |
| H | -1.647898 | 3.478415 | -6.457428 |
| H | -3.021321 | 3.313335 | -7.681199 |
